# Self-organized spectral adjustment in echolocation sounds within group-flying bats

**DOI:** 10.1098/rspb.2026.0921

**Published:** 2026-10-14

**Authors:** Kazuma Hase, Tasuku Miyazaki, Seiya Oka, Noriyoshi Senoo, Hiraku Nishimori, Masashi Shiraishi, Ken Yoda, Kohta I. Kobayasi, Shizuko Hiryu

**Affiliations:** ^1^ Department of Systems Function and Morphology, University of Toyama, Toyama, Japan; ^2^ Department of Psychology, Neuroscience & Behaviour, McMaster University, Hamilton, Ontario, Canada; ^3^ Graduate School of Environmental Studies, Nagoya University, Nagoya, Japan; ^4^ Faculty of Life and Medical Sciences, Doshisha University, Kyoto, Japan; ^5^ Meiji Institute for Advanced Study of Mathematical Sciences, Meiji University - Nakano Campus, Nakano, Tokyo, Japan

**Keywords:** acoustic interference, bat, collective behaviour, echolocation, self-organization

## Abstract

Self-organized behaviour has been extensively studied in group dynamics in animals, with a strong focus on movement coordination in species like fish and birds. By contrast, far less attention has been paid to vocal interactions within animal groups. Here, we unveil a self-organized vocal behaviour in echolocating bats (*Miniopterus fuliginosus*), characterized by their adaptive responses to jamming stimuli. Using controlled playback experiments, we identified a mathematical model that describes how bats autonomously adjust their vocal frequencies to avoid signal interference mimicking group echolocation. The model was subsequently extended to simulate a group of bats flying in two-dimensional space. Consistent with previous observations of group-flying bats, the simulation revealed frequency-adjusting behaviour, characterized by an overall increase in frequency differences within the group. Crucially, we demonstrated that this behaviour reduces potential misdetections during echolocation, enhancing group-wide sensory accuracy. These results reveal a mechanism of conflict resolution through self-organized vocal adjustment and establish a parallel with other self-organizing systems in nature. Our findings reveal that this frequency-adjusting behaviour represents a self-organized vocal behaviour that effectively mitigates conflicts within a group, one achieved via simple behavioural rules, as in other types of collective animal behaviour.

## 1. Introduction

Self-organization is a phenomenon whereby the interaction among individuals generates order at the level of a group. In animals, cooperative group behaviour emerges when individuals follow several simple rules. For instance, in a school of fish [1] or a flock of birds [2,3], each individual determines its movement based on its position relative to the others, resulting in a coordinated movement of the group as a whole. Studies of such group behaviour have primarily focused on how the movement of individuals is governed depending on their positional relationship to others within the same group [4]. On the other hand, unlike fish and birds that rely on passive sensing, echolocating bats perceive the world by comparing self-emitted ultrasounds with the returning echoes [5], enabling these mammals to track small flying insects in total darkness [6]. In order to avoid collisions with others and/or capture prey, echolocating bats may need to mitigate potential acoustic interference that may be caused by signals emitted by other members for sensing. Within a group, each bat is exposed to the other bats' vocalizations, which may impair their respective echolocation ability [7,8].

It is known that bats can change the acoustic characteristics of their echolocation pulses in the presence of other individuals or jamming stimuli. For example, in the field, *Tadarida brasiliensis* flying in a pair emitted pulses whose terminal frequency (TF) differed [9]. Similarly, in the laboratory, when flying in a group of four, *Miniopterus fuliginosus* bats shift their TF so that the mean TF differences increase in the group [10]. Importantly as well, these frequency shifts can reduce the cross-correlation-based similarity indices of pulses of bats within a given group [10]. These types of frequency-shifting behaviour are consistent with jamming avoidance responses analogous to those in weakly electric fish [11], although other acoustic characteristics such as duration [12,13], sound pressure levels [10,13,14] and interpulse intervals [15–17] can change under acoustic jamming.

When multiple bats are flying together, even in only a group consisting of relatively few individuals, each bat in the group would experience an ever-changing acoustic scene created by the other bats' vocalizations. If the frequency-shifting behaviour is a jamming avoidance response, how does each bat in the group decide whether, and how, to alter their respective frequency (and of course other acoustic characteristics in tandem) in response to the complex dynamic acoustic scene? Here, we hypothesize that frequency adjustments across an entire group could be autonomously achieved by simple behavioural rules that each member follows.

Here, we conducted behavioural experiments to investigate how bats flying alone modified their own TFs in the presence of jamming stimuli composed of different TFs, interpulse intervals (IPIs) and sound pressure levels (SPLs). We hypothesized that changing frequency, IPIs and SPLs could mimic overall acoustic interference caused by different group sizes. Through this simple experiment, we identified several behavioural rules for bats' frequency adjustment. Specifically, bats shifted their TFs depending on the IPIs and SPLs of the jamming stimuli. Furthermore, we observed an upward shift in the TF response to stimuli when the jamming TF was lower than their own TFs. Conversely, when the jamming TF was higher, their TFs shifted slightly downward or remained unchanged. Then, we found that these simple behavioural rules in the presence of the jamming stimuli can be described and predicted by the leaky-integrator model. By integrating incoming signals over time while accounting for a decay (or leak), this model can capture various biological phenomena such as membrane potentials of a neuron [18], decision-making in animals [19] and vocal amplitude adjustments in echolocating bats [20]. Furthermore, by conducting simulations of agent bats flying in a group using this model, we successfully replicated the frequency-adjusting behaviour observed in our previous behavioural study [10]. Altogether, our results demonstrate that frequency-adjusting behaviour in bats flying in a group can be explained by relatively simple, self-organized behavioural rules. In this model, bats do not rely on a fine temporal and spectral resolution of acoustics. Instead, conflicts within the group can be mediated by individuals following simple behavioural rules, similar to other types of collective behaviour, such as bird flocking and fish schooling.

## 2. Results

To investigate how bats changed the acoustic characteristics of their emitted echolocation pulses in response to different levels of acoustic jamming, we presented solitary flying bats with jamming sounds that mimicked frequency modulation (FM) echolocation pulses having different IPIs (figure 1a). Echolocation pulses emitted by flying bats were recorded by a miniature telemetry microphone attached to each bat's back [21]. The jamming stimuli consisted of repetitive FM jamming sounds, whose bandwidth was 40 kHz with a 3 ms duration, and the temporal change of their frequency mimicked their downward FM echolocation pulses [22,23]. We generated two types of jamming stimuli, with low versus high TFs compared with the overall mean TFs of *M. fuliginosus*. The bandwidth was fixed to 40 kHz, rendering the overall shapes the same across conditions.

**Figure 1.**
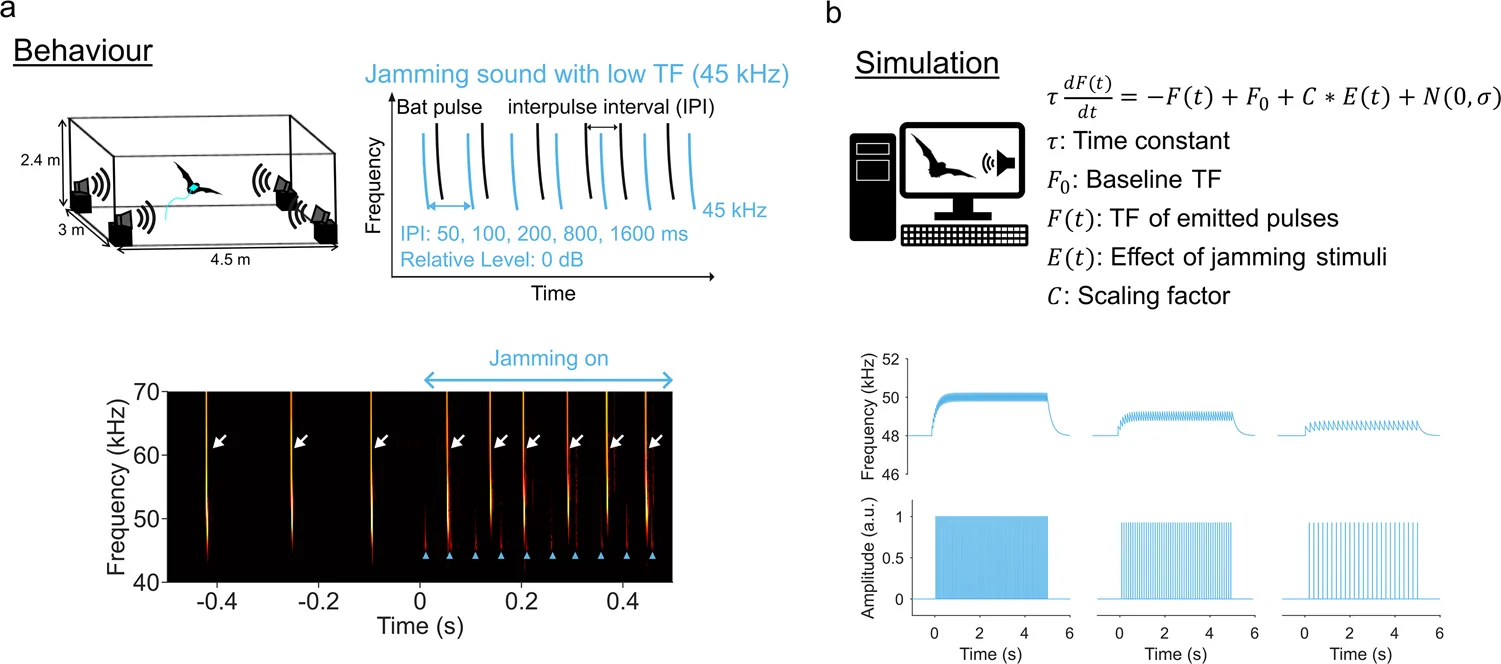
Schematic illustration of the study. (a) Behavioural experiments. (Top left) Experimental setup. Bats carrying a telemetry microphone flew alone in a flight space where four loudspeakers were located at the corners. They were exposed to jamming stimuli presented from the speakers. (Top right) Schematic spectrograms of jamming stimuli with low terminal frequency (TF, blue) in comparison with the bats' emitted pulses (black). (Bottom) Example spectrogram of telemetry recording showing pulses emitted by the bat (white arrows) and jamming stimuli presented via the speakers (blue triangles). (b) Mathematical simulation. (Top) A leaky-integrator model to reproduce changes in TF observed in the behavioural experiments. (Bottom) The model outputs in response to different interpulse intervals (IPIs) of jamming stimuli. Panels in the upper row show frequency changes across time calculated based on the model. Panels in the lower row represent the input function *E*(*t*) with IPIs of 50, 100 and 200 ms.

TF is overrepresented in the auditory system of FM echolocating bats [24,25] and auditory sensitivity is highest around TF in *M. fuliginosus* [26]. Moreover, removing frequency around TF in virtual echoes deteriorates target ranging performance in *Eptesicus fuscus* [27]. Our previous observations demonstrate that group-flying bats increase inter-individual TF differences but not other acoustic characteristics such as initial frequency, bandwidth, duration and sound pressure level [10]. Thus, here we exclusively focused on how echolocating bats modify TF depending on the temporal density of FM jamming sounds and whether and how the behavioural responses contribute to TF variations in group-flying bats.

### (a) Leaky-integrator model reproduces terminal frequency shifts to various jamming stimuli

Bats shifted the TFs of their emitted pulses upward in response to jamming stimuli with a low TF, and these upward shifts increased as the IPIs of jamming stimuli decreased (ANOVA: *F*_4,30_ = 11.6, *p* < 0.05; figure 2a–c; supplementary material, figure S3, left; results for other acoustic characteristics can be found in supplementary material, figures S1 and S2). The shifts were significantly different between shorter and longer IPIs (respectively, 50 ms vs 200, 800 or 1600 ms, and 100 ms vs 800 or 1600 ms; Tukey–Kramer, all *p*-values <0.05; figure 2c). Whereas the mean TF shift was 1.4 ± 0.8 kHz when IPI of the jamming stimuli was the shortest (IPI: 50 ms), it was much smaller, 0.0 ± 0.2 kHz, in the presence of jamming stimuli with the longest IPI (IPI: 1600 ms). Because the IPI of jamming stimuli, which can be considered as intervals of sounds among multiple bats, can represent the density of animals in a group, these results demonstrate that echolocating bats can shift their TF according to group size.

**Figure 2.**
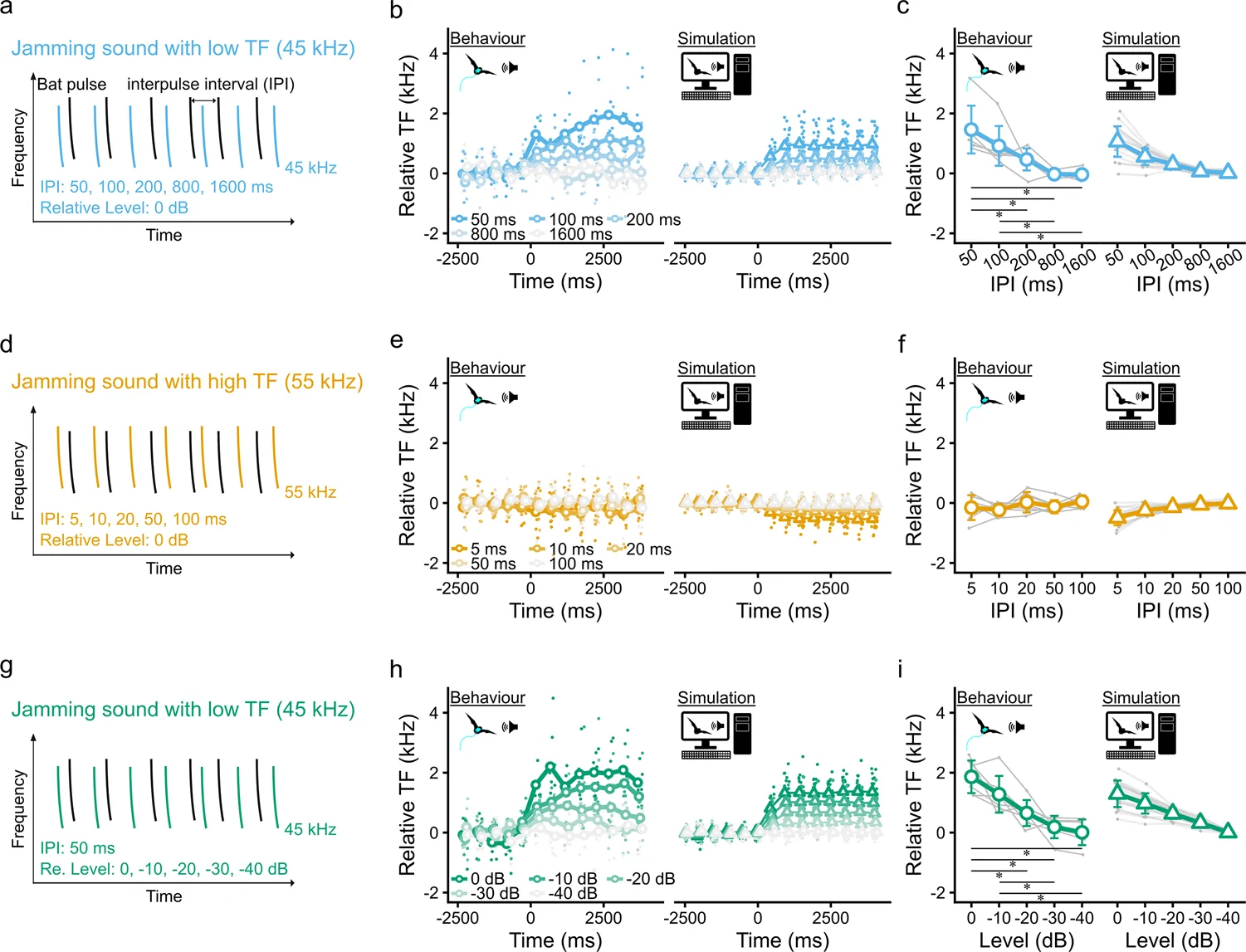
Behaviour and modelling of terminal frequency (TF) shifts in response to different jamming stimuli. (a,d,g) Schematic diagrams of the jamming stimuli: low-TF stimuli with different inter-pulse intervals (IPIs; a,g) and high-TF stimuli with different IPIs (d). In (g), low-TF stimuli were presented at different sound pressure levels (SPLs). (b,e,h) Time courses of TF shifts in behaviour (left) and in simulation (right) in response to the corresponding stimuli. Symbols show individual mean TFs, and solid lines indicate population means. (c,f,i) Mean TF shifts in behavioural and simulated data across conditions. Bars indicate mean ± s.d. Asterisks denote statistical significance (*p < 0.05).

Next, we examined how jamming stimuli with a high TF may have affected the acoustic characteristics of echolocation pulses. With respect to *M. fuliginosus*, our recent study revealed that they did not change their TF in response to jamming stimuli that had a TF higher than their own [28]. Also, it has been observed that some individuals of that species shifted their TF downward when flying in a group [10]. Since we detected here upward shifts caused by jamming stimuli with a low TF that depended on the IPIs of stimuli, we hypothesized that high-TF jamming stimuli with much shorter IPIs could elicit downward shifts in the bats' TF. To test this, we used high-TF jamming stimuli with shorter IPIs (5, 10, 20, 50 and 100 ms, figure 2d) than those used for low-TF jamming stimuli. Overall, the high-TFjamming stimuli failed to induce significant shifts in TF (ANOVA: *F*_4,30_ = 1.2, *p* = 0.3153; figure 2e,f; supplementary material, figure S3, middle). The mean TF shift was −0.2 ± 0.4 kHz in response to the shortest IPIs (−0.2 ± 0.4 kHz; one sample *t*-test: *p* = 0.3632). However, two of seven tested bats exhibited slightly decreased TFs under extreme conditions (please refer to bats 601 and 603 in supplementary material, figure S3, middle). This latter result implied bats might change their TFs bi-directionally and asymmetrically according to spectral relationships between their own TF and the TFs of surrounding jamming stimuli.

Another factor by which to gauge acoustic jamming is SPLs, so we then exposed bats to jamming stimuli with differing SPLs. In this experiment, only a low-TF jamming stimulus was applied, given the above negligible effects of high-TF jamming stimuli on TF. Evidently, those observed upward shifts in the TFs of bats' emitted pulses decreased as the SPLs decreased (ANOVA: *F*_4,30_ = 17.64, *p* < 0.05; figure 2g–i,f; supplementary material, figure S3, right). Notably, the shifts differed significantly in magnitude among SPLs (0 dB vs −20, −30 or −40 dB, and −10 dB vs −30 or −40 dB; Tukey–Kramer: all *p*-values <0.05; figure 2i). The bats did not shift TFs in response to jamming stimuli when the stimuli were presented at −40 dB relative to the maximum SPL (mean TF shift: 0.0 ± 0.4 kHz).

### (b) Self-organized terminal frequency shifts reduce acoustic jamming within a group

The IPIs of jamming stimuli can indicate the number of or the density of individuals in a group, while the SPLs of jamming stimuli can be used to roughly gauge the distance among group members. Also, because every bat in a group receives pulses from other members entailing inter-individual variation, the TFs of jamming stimuli are related to the relation of TFs among individuals of that group. Our previous study suggests that *M. fuliginosus* shifts TF most when the jamming TF and the shifts decreased as the jamming TF increased [28]. These findings led us to formulate the following hypothesis. First, the bat with the highest TF in a group is most affected by other individuals because they all have lower TFs than it, so it responds and changes its TF the most; likewise, the bat having the second highest TF is the second-most affected and so its change in TF should be the second greatest in magnitude; conversely, the bat with the lowest TF is least affected, hence its TF is changed the least in magnitude. In this way, as a group, these bats could achieve a frequency-adjusting behaviour autonomously, in the absence of any central command (figure 3).

**Figure 3.**
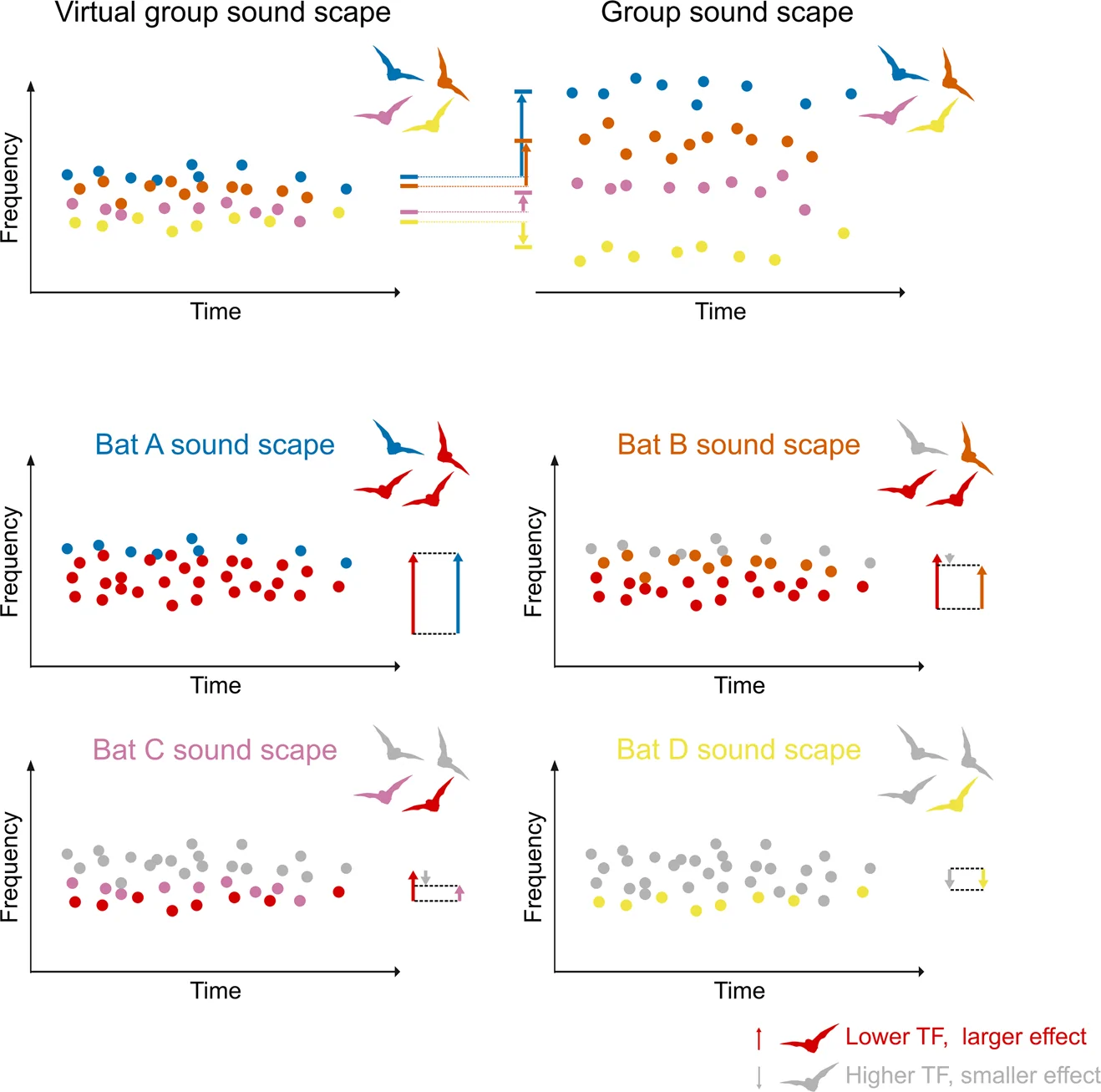
Schematic illustration of the proposed mechanism underlying frequency adjustment during group flight. Each bat in a group of four shifted its terminal frequency (TF) according to different levels of acoustic interference, according to the spectral relationship to the pulses of the other bats. The bat with the highest TF (Bat A) is most affected by the other bats and thus shows the largest TF shifts. By contrast, the bat with the lowest TF (Bat D) is least affected by the others and exhibits subtle downward TF shifts. As a result, shifts in TF differ across individuals, leading to self-organized spectral adjustments as a group.

To test that hypothesis, we constructed a mathematical model describing changes in TF in response to jamming stimuli, and then applied the model to more realistic situation, that is, group flight (see §4g, ‘Modelling the frequency-shifting behaviour in response to jamming stimuli’ in Material and methods). We described the bat responses to jamming stimuli via a leaky-integrator model (figure 1b). A leaky integrator is a simple dynamical system that accumulates incoming inputs while continuously losing part of the accumulated value over time, thereby retaining a short-term memory of recent inputs rather than responding only to the most recent stimulus. The model captures temporal dynamics of biological phenomena from membrane potentials of a neuron [18] to vocal amplitude control in echolocating bats [20], by integrating incoming signals over time while accounting for a leak. Our leaky-integrator model worked as follows. First, the input was a rectangular pulse train representing the overall power of each jamming stimulus. The model then integrated the pulse train with a leak, so that the effect of successive jamming stimuli accumulated over time while the accumulated signal gradually decayed in the absence of stimulation, reflecting the basic nature of frequency-shifting behaviour of bats where they rapidly shift frequency in response to jamming sounds and frequency returns to the baseline after the sound is turned off [29]. Next, the TF was determined by multiplying the integrated signal by a scaling factor whose value depended on differences in the TFs between a bat and a jamming stimulus.

Overall, the model gave results similar to those observed in the behavioural experiments (figure 2). The modelled bats shifted their TFs upward in response to low-TF jamming stimuli, but these shifts decreased as the IPIs of jamming stimuli increased (figure 2b,c). The modelled bats did not modify their TFs in response to high-TF jamming stimuli, though we discerned slight downward shifts under the shortest IPI condition (figure 2e,f). Importantly, the upward shifts in the TFs of modelled bats in response to low-TF jamming stimuli decreased as the SPLs decreased (figure 2h,i). Although direct statistical comparison was difficult because of differences between our model and the experimental setting, the model qualitatively reproduced fundamental behavioural features observed in bats responding to acoustic interferences during simulated group flight.

Then, the model was extended to simulate the behaviour of group-flying bats. We first generated flight trajectories of four bats in a 5 × 5 m space. Inter-individual differences in the distance and the angle in the generated trajectories were used to calculate the effect of the atmospheric attenuation and directionality of emitted pulses. Each individual had its own TF as an initial value, generated from a normal distribution. The modelled bats emitted pulses with IPIs determined by a gamma distribution (supplementary material, figure S4a). For the sake of simplicity, the bats emitted pulses at a constant SPL. We estimated the SPLs of pulses that one bat received from co-occurring bats by considering atmospheric attenuation and directionality of emitted pulses, which were calculated based on the distances and angles among individuals (supplementary material, figure S4b,c).

Figure 4 shows an example flight trajectory and changes in TFs of the real and simulated single and group flights of four bats. The graphs showing the data of real bats flying in groups were adapted from our previous study [10]. Real bats showed broader differences in their TF in group flight than in single flight (figure 4a–d). Telemetry recordings from each individual of four bats demonstrated increased individual differences in their mean TFs in group flight in comparison with those in single flight (figure 4i) [10]. Similarly, the simulated group flights (figure 4e–h) showed that the individual differences in mean TFs rose from 0.6 ± 0.6 [95% confidence interval (CI): 0.54–0.72] kHz and 0.7 ± 0.6 [0.57–0.75] kHz in single flights 1 and 2, respectively, to 1.3 ± 0.7 [1.18–1.41] kHz in the group flight (figure 4j). The same simulations were performed with a group size of 8, 16 and 32 bats (figure 5a–d). This revealed a similar pattern in the larger groups (figure 5e). The frequency differences among individuals were 0.3 ± 0.3 [0.34–0.41], 0.2 ± 0.2 [0.20–0.23] and 0.1 ± 0.2 [0.12–0.14] kHz in single flight 1, but increased to 0.9 ± 0.6 [0.79–0.92], 0.5 ± 0.5 [0.45–0.52] and 0.3 ± 0.4 [0.26–0.30] kHz in the group flight for group sizes of 8, 16 and 32, respectively.

**Figure 4.**
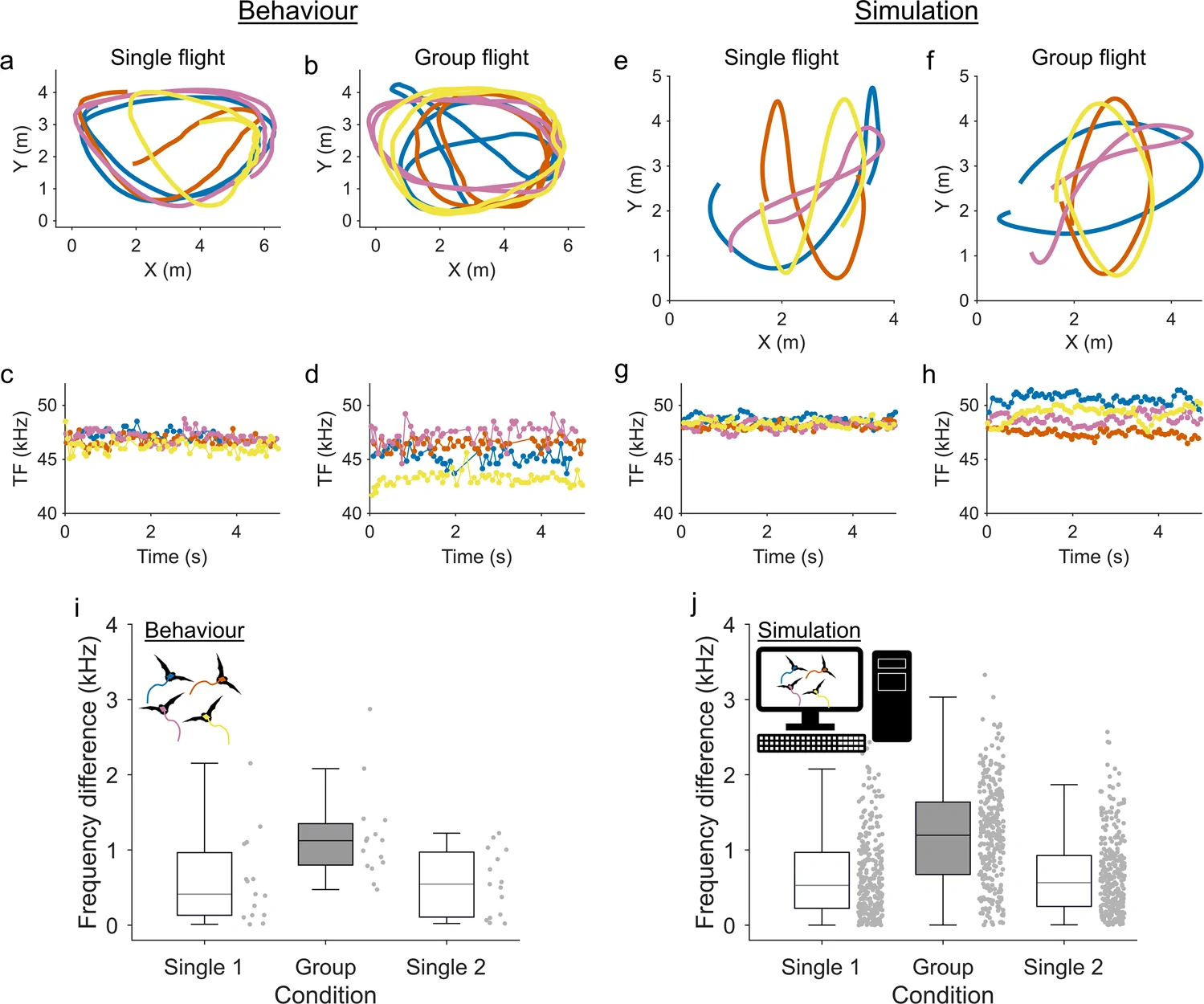
Behaviour and modelling of terminal frequency (TF) shifts in a group of four bats. (a,b) Examples of flight trajectories of four bats during single (a) and group flights (b). Colours represent different individuals. (c,d). Corresponding time course of TF emitted during single (c) and group flights (d). (e–h) Examples of simulated flight trajectories of four bats during single (e) and group flight (f) and corresponding time courses of TFs (g,h). (i,j) Summary of changes in inter-individual differences in mean TFs across different flight conditions observed in behavioural experiments (i) and in simulations (j). Data in (a–d,i) were replotted from previously published data (10).

**Figure 5.**
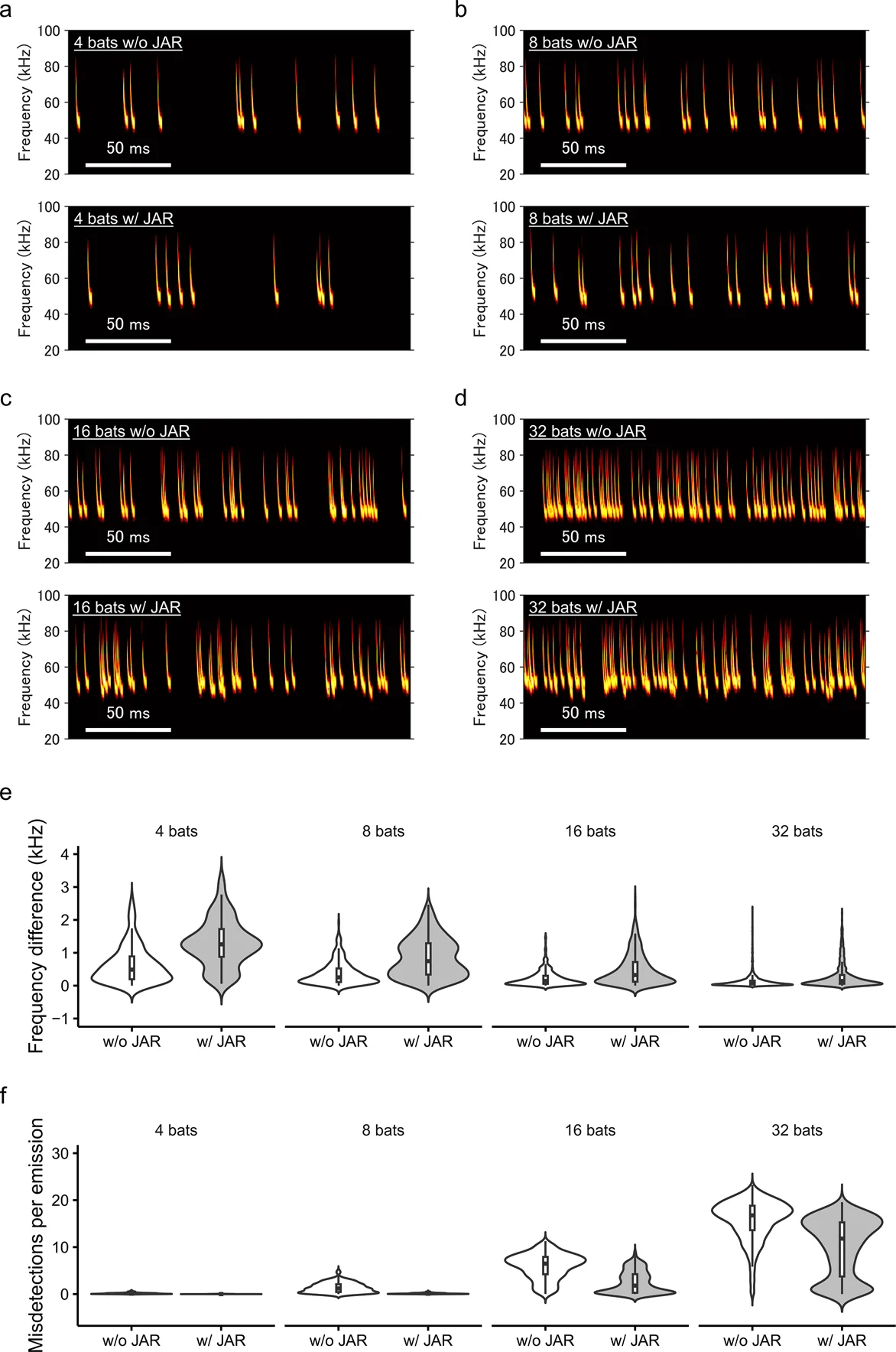
Relationship between bats' group size and frequency-shifting behaviour. Examples of spectrograms of pulses a bat receives from the other group members in each simulation for a group size of four (a), eight (b), 16 (c) or 32 (d) bats. (e) Frequency differences among individuals in a group, with (w) and without (w/o) jamming avoidance response (JAR). (f) Tallied possible misdetections in each group flight, with and without JAR. Violin plots indicate kernel densities of each variable for a different group size and the presence/absence of JAR.

Lastly, to estimate the effect of jamming avoidance responses (JARs) on echolocation performance, we tallied the number of possible misdetections that occurred in the above simulations of group-flying bats (figure 5f). First, we calculated the cross-correlation function between each pulse and other bats' sounds detected in a given time window (each spanning the period from current pulse emissions to the next pulse emissions). For this estimation, we normalized all the sounds so they had the same amplitude, rendering the cross-correlation values to range from 0 to 1. The modelled bats were presumed to detect pulses when the peaks of cross-correlation values exceeded 0.5 (supplementary material, figure S6). The frequency differences among group members decreased as the group size increased, and they were larger in the simulated groups where each individual employed JARs (figure 5e). The number of possible misdetections fell from 0.14 ± 0.14 to 0.01 ± 0.03 for the four-bat group, from 1.37 ± 1.07 to 0.12 ± 0.12 for the eight-bat group, from 5.98 ± 2.67 to 2.49 ± 2.33 for the 16-bat group, and from 15.77 ± 4.34 to 10.01 ± 6.11 for the 32-bat group, when JARs were employed by all members (figure 5f).

## 3. Discussion

Here, by using echolocating bats as a study species, we examined how their self-organized vocal behaviour may reduce acoustic interference that could arise during group flight [8,30]. We found that frequency adjustment could be described by a leaky-integrator model, which was then extended to simulate interactions among multiple bats. The simulations suggest that the self-organized frequency-adjusting behaviour increases inter-individual frequency differences while reducing potential acoustic interference. Together, these findings demonstrate how simple behavioural rules can give rise to self-organized frequency-adjusting behaviour in group-flying bats.

Leaky-integrator models have been used to study decision-making in noisy environments by vertebrates such as zebrafish [19] and primates [31], and vocal control in echolocation by bats [20,32]. A leaky-integrator model can explain well the involuntary increases in the amplitude of pulses emitted by resting *E. fuscus* in response to broadband noise (i.e. the Lombard effect), demonstrating that the amplitude can be adjusted within 30 ms from the start of the noise presentation. These findings suggest that the Lombard effect and frequency-shifting behaviour share similar mechanisms of audio-vocal integration involving processes achieved by a leaky integrator.

Horseshoe bats (*Rhinolophus ferrumequinum*) compensate for Doppler shifts in echo frequency by lowering their pulse frequency to maintain an echo frequency within a narrow frequency range, called the reference frequency, where their auditory sensitivity and frequency resolution are extremely high [21,33,34]. Over 20 years ago, Metzner *et al.* [35] introduced a conceptual audio-vocal feedback model to explain how bats achieve the Doppler shift compensation. We propose a similar model wherein the pulse frequency is determined by integrating the power of a jamming stimulus with some leak in auditory nuclei, with the inputs (or outputs) to the nuclei scaled by the frequency of the stimuli. The model simply relies on the spectral information and does not require extraordinary spectro-temporal resolutions in the auditory system. Also, the model realizes self-generated frequency adjustments without higher-order processes, such as decision-making, to mitigate acoustic jamming in a group of flying bats.

The present model does not have prediction-related processing. Because sensory error-driven vocal frequency adjustments have been suggested to be a shared mechanism for fine control of vocal frequency in both humans and echolocating bats [36,37], incorporating sensory prediction-related components into the current framework may provide further insights into how echolocating bats adjust their vocalizations based on sensory feedback under acoustic interference.

We found that two out of seven tested bats showed downward shifts in their TFs in response to high-TF jamming stimuli with shorter IPIs. Several studies have shown that bats shift their TF downward in response to jamming stimuli [38,39], and our own previous work also suggests that some individuals decrease their TF in a group of four bats [10]. Here, we observed notable downward shifts in TF in the simulation as in a real group of flying bats [10], probably because we included asymmetrical effects from other bat pulses having a lower TF and those having a higher TF (supplementary material, figure S3, middle). Because downward shifts are uncommon in FM echolocating bats in response to conspecifics jamming, we suggest that echolocating bats are affected by the presence of pulses whose TFs are higher than their own TFs, and the resulting effect can sometimes be discerned as downward shifts in TF when that effect is strong enough, contributing to self-organization of TF differences among groups. Further detailed investigation is needed to understand how jamming stimuli with higher TF affect the behaviour of bats and also their internal state.

What is the possible functional role of TF shifts? One of the simplest hypotheses is that bats diverge in emitted frequencies to avoid spectral overlapping of their own echoes with other sounds from co-occurring bats. It has been demonstrated that a slight shift in frequency sweeps of a bat-like signal causes a significant decrease in the cross-correlation peak [10]. Indeed, our simulation reveals that the number of misdetections is consistently reduced during the frequency-shifting behaviour (figure 5). The consistent reduction in misdetections indicates that JAR improves signal separability, although the absolute number of competing signals remains high. It is important to note that the absolute number of misdetections may be overestimated in the current model, as we manipulated only the TF of pulses while keeping other acoustic parameters constant and counted all pulses until the next emission. Furthermore, our analysis was based on the absolute number of overlapping pulses rather than a signal-to-background metric. Incorporating signal-to-background ratio or other perceptually relevant measures into future models may reduce the estimated number of false alarms while providing a more biologically realistic assessment of the benefits of frequency shifting.

Ultrasonic pulses transmitted from a bat to other conspecifics are attenuated in a way that depends on differences in the distances and angles between these bats [40–42]. Moreover, bats might process only sounds occurring within a short time window after they emit a pulse [43,44]. Moreover, inter-individual differences in pulse characteristics can also influence the similarity of sounds among members in a group [13,45]. In the present study, we assumed that playback stimuli elicited frequency-shifting responses comparable to those evoked by conspecific echolocation calls, allowing us to isolate the effect of terminal frequency differences on jamming avoidance. However, bats might use inter- and intra-individual natural fluctuations in acoustic characteristics of emitted pulses to solve acoustic interference. Thus, it must be investigated in the future whether and how echolocating bats respond to jamming stimuli with those natural variations and how they are related to the present model. Furthermore, bats are known to alter the duration [10,12,13], amplitude [10,13,28] and interpulse interval of pulses in response to sounds of conspecifics [15,16,46,47]. Thus, those passive and active variations in pulse acoustic characteristics will lessen the number of misdetections, which can be further reduced with the self-organized frequency-adjusting behaviour, enabling bats to echolocate in a large group.

We found that the number of misdetections decreased with JAR but remained relatively high in the groups of 16 or more bats. The present simulation focused on the shifts in frequency while keeping the overall spectro-temporal characteristics. We must mention that this may have overestimated misdetections because inter- and intra-individual variations in echolocation pulses are suggested to effectively reduce signal similarity among individuals in a group [13,45]. Also, the rather large number of misdetections may be because all the bats stayed in a confined space in our simulation, which is not a realistic assumption in the wild, even though *M. fuliginosus* lives with thousands of individuals in a cave. It is reported that bats spatially spread after emerging from the cave and jamming avoidance responses are not necessary in low-density groups [48]. The present findings demonstrate that spectral jamming avoidance-like behaviour can be realized by simple behavioural rules, which can be effective for relatively small group sizes, probably fewer than 10 bats.

How do echolocating bats navigate under severe jamming in natural group flight? Notably, bats can still detect nearby conspecifics under strong acoustic interference even in large groups consisting of 100 individuals [49], although frequency-dependent mechanisms were not explicitly examined in the study. However, spectral jamming avoidance alone may not fully resolve acoustic interference. Recent computational studies have shown that spectral jamming avoidance may have limited effectiveness in reducing acoustic interference [48,50]. These studies assume linear frequency chirps as bat pulses, which are relatively robust to frequency shifts compared with hyperbolic and logarithmic sweeps [10]. Frequency shifts in naturalistic echolocation calls reduce but not eliminate conspecific or heterospecific jamming, implying that additional factors may contribute [51]. Together with passive acoustic effects and other behavioural adjustments, frequency shifting may therefore partly contribute to reliable echolocation during natural group flight.

## 4. Material and methods

### (a) Subjects

A total of 11 *Miniopterus fuliginosus* (six males and five females) were used in this study, and seven bats were tested for each experimental condition. They were caught from wild colonies in caves in Fukui Prefecture, Japan. We were licensed to collect these bats and carried out the study in compliance with all Japanese laws (permits from Fukui Prefecture in 2020, 2021 and 2022). The bats were housed in a temperature- and humidity-controlled room (4 (L) × 3 (W) × 2 m (H)) at Doshisha University, Kyoto, Japan. Food (mealworms) and water were made available ad libitum to the bats. The day/night cycle of the room was set to 12 h/12 h. All experiments were conducted in accordance with all Japanese laws. All experimental protocols were approved by the Animal Experiment Committee of Doshisha University (permission nos: A20026, A21030 and A22015).

### (b) Experimental design

The experimental procedures used followed those applied in previous studies [12,22,28]. Recordings were made in an experimental chamber (9 × 4.5 × 2.4 m) at Doshisha University, Kyoto, Japan. This experimental chamber was constructed with steel plates to reduce external electromagnetic noise and signals from commercial FM radio stations. Its inside walls were lined with sound-attenuating foam, to reduce acoustic reflections. A telemetry microphone [21,52] was attached to the back of a bat to record its emitted pulses in flight (for the details, please refer to the following section (§4c)). During the experiments, long-wavelength lighting with filters (removing wavelengths <650 nm) was employed to prevent a bat from using visual cues [53]. Solitary bats were flown in a flight space (3.0 × 4.5 × 2.4 m) delimited by a net suspended from the ceiling. Four loudspeakers (Pioneer, PT-R7 III, Kanagawa, Japan) were set at a fixed height (1.4 m) in each corner of the flight space (within the chamber).

We first flew a bat in the flight space for approximately 20 s, and then exposed it to a jamming stimulus (5 s) while flying; after the presentation of the jamming stimulus ended, the bat kept flying for another 20 s. During this procedure, none of the 11 bats landed on the walls, the ceiling or the floor, though some bats did occasionally try to land somewhere in the flight space. We considered the 5 s period before the presentation as the ‘jamming off’ condition, and the 5 s period of the presentation of jamming stimulus as the ‘jamming on’ condition. We tested seven bats once for each of the three different stimuli.

### (c) Sound recordings

Echolocation pulses emitted by a bat during flight were recorded with a miniature telemetry microphone [52]. It consisted of a 1/8 inch omnidirectional electret condenser microphone (Knowles, model FG-23329-C05, Itasca, IL, USA) and a custom-made amplifier and transmitter circuit with a transmitting antenna, powered by a 1.5 V hearing-aid battery (Sony, type SR626SW or SR726SW, Tokyo, Japan). Including the battery, the telemetry microphone weighed approximately 0.7–0.9 g, this being just 3 to 7% of a bat's weight, and thus easily carried when it flew [10]. Just before its flying trial, to the back of each bat, we gently attached the telemetry microphone with a piece of double-sided adhesive tape; then, after completing each experiment, we carefully removed the microphone with a parting agent (Toagosei, Aron Alpha Specific Peeling Liquid, Tokyo, Japan).

The telemetry microphone transmitted FM radio signals having a carrier frequency between 76 and 104 MHz, depending on the resonant frequency of the circuit. These signals were received with an FM radio antenna (Terk Technologies Corporation, FM+, Commack, New York, USA) suspended from the ceiling and demodulated and then band-pass filtered (10–200 kHz) with a custom-made multi-channel FM receiver (ArumoTech Corporation, Kyoto, Japan). The de-modulated signals were then digitized with a USB data acquisition device (16 bit, sampling frequency *f*_s_ = 500 kHz; model NI USB-6346 or model NI USB-6356, National Instruments, Austin, TX, USA). The frequency response of the telemetry microphone system was flat (within ±3 dB) between 20 and 100 kHz [10].

### (d) Jamming stimuli

Jamming stimuli consisted of sequences of downward FM signals differing in terminal frequency (TF), sound pressure levels (SPLs), or interpulse intervals (IPIs). These FM signals, designed to mimic bats' echolocation pulses according to previous studies [22,23], were 3 ms in duration and 40 kHz in bandwidth. We prepared two types of FM signals in terms of TF. One was modulated from 85 to 45 kHz (‘low TF’) and the other from 95 to 55 kHz (‘high TF’), determined in relation to the mean TF of *M. fuliginosus* flying in the chamber (approximately 48 kHz). To understand how bats respond to different IPIs of the jamming stimuli with contrasting TFs, we generated the low-TF stimuli with IPIs of 50, 100, 200, 400, 800 and 1600 ms and the high-TF stimuli with IPIs of 5, 10, 20, 50 and 100 ms. A different set of IPIs for low and high-TF stimuli was used because we hypothesized the high-TF stimuli, which usually did not induce shifts in TF [28], would cause shifts in the TFs of pulses emitted by bats if they were presented with higher repetition rates. The IPIs of the low-TF jamming stimuli were selected based on our previous findings in the same species, in which they show robust frequency shifts in response to the jamming IPIs of 35–50 ms [22,28]. We used much shorter IPIs for the high-TF jamming stimuli to make the situation more severe because our previous and preliminary observations suggested that the stimuli with IPIs around 35–50 ms did not evoke clear shifts in TF [28]. The SPLs used here were 0, −10, −20, −30 and −40 dB (relative to the maximum SPL) for both low-TF and high-TF stimuli with an IPI of 100 ms. Because the SPL was fixed within each condition, variation of the SPLs occurred depending on the spatial relationship between a bat and the loudspeakers. Estimated maximum sound pressure levels of freely flying bats in the field range from 120 to 130 dB SPL for open space foragers like *M. fuliginosus* [42,54]. Although we aimed for those SPLs, the maximum SPL of the jamming FM signals was set at 110 dB SPL, peak-to-peak, at 0.1 m from each loudspeaker, which was the limit for the loudspeaker in the frequency range of the jamming stimuli.

### (e) Sound analysis

Echolocation sounds emitted by the bats were manually analysed from sound spectrograms of the telemetry microphone recordings, using custom-written MATLAB scripts on a personal computer. When jamming signals were simultaneously recorded with the telemetry microphone, echolocation sounds were carefully selected based on the difference in spectro-temporal patterns between pulses and jamming signals. We defined the TF as the lowest frequency in the spectrograms (256-sample Hanning window with 95% overlap and a 2048-point Fast Fourier transform (FFT)) that was −25 dB from the maximum energy portion of each pulse. The IPIs and their duration were also determined from the spectrograms at −25 dB relative to the maximum energy portion of each pulse. Although second-harmonic-based clutter rejection has been demonstrated in *E. fuscus* [55], there is currently no evidence that *M. fuliginosus* uses second-harmonic information in this manner. Therefore, we focused on the first harmonic in the present study.

### (f) Statistical analysis

We used linear mixed models (LMMs) to test whether the TF, pulse duration or initial frequency (IF) of echolocation pulses was affected by the IPIs or SPLs of the jamming stimuli. We built separate LMMs for three different conditions (i.e. IPI conditions with a low or high TF and SPL condition with a low TF). The response variables were changes in the TFs, durations or IFs of sounds emitted by the bats. The fixed effects were the variable (IPIs or SPLs), the presence (jamming off or on) of the jamming stimuli and the interaction of these two effects. The bat ID was included as a random effect. In the case of a significant main effect or interaction, *post hoc* tests were performed using the Tukey–Kramer method to adjust the type I error control to correct for the number of multiple comparisons.

The model fit was quantified by plotting residuals. A type II Wald $\chi^{2}$-test was used to detect significance of the fixed effects. In the case of a significant main effect or interaction for either, *post hoc* tests were performed using the Tukey–Kramer method to adjust the type I error control to correct for the number of multiple comparisons.

All statistical analyses were implemented in the R platform (v. 4.3.0) [56]. We used the lmer function of the ‘lme4’ package (v. 1.1.33) for building LMMs [57], and the Anova function of the ‘car’ package (v. 3.1.2) for the type II Wald $\chi^{2}$-tests [58]. The model fit was quantified by using the function simulateResiduals of the ‘DHARMa’ package (v. 0.4.6) [59]. The emmeans function of the ‘emmeans' package (v. 1.8.6) was used to perform the *post hoc* comparisons [60]. *p*-values <0.05 were considered significant. Results are presented as mean ± s.d., unless otherwise stated.

### (g) Modelling the frequency-shifting behaviour in response to jamming stimuli

The present study observed integration-like behavioural responses where shifts in TF changed dependently on the IPI of the jamming stimuli. Also, after the jamming stimuli were turned off, it is known that TF returned to the baseline within several hundreds of milliseconds [12,28]. These findings led us to hypothesize that a leaky-integrator model could reproduce dynamics of TF changes over time in response to dynamics by integrating inputs over time with a leak [19,20]. A leaky-integrator model was used to simulate the frequency-shifting behaviour by a flying bat in response to the jamming stimuli, using this equation:

$$\tau \;\frac{dF(t)}{dt}=F_{0}\;-F(t)+CE(t),$$

where τ is the leak time constant of 200 ms, which corresponds to the rapidness of spectral changes of echolocation pulses in response to jamming sounds ranging from approximately 60 to 300 ms in previous studies [22,28,38,61]; $F_{0}$ is the baseline TF of the bat; F(t) is the TF of emitted pulses by the bat; E(t) is the effect of jamming stimuli represented by rectangular pulses whose amplitudes is equal to those of the jamming stimuli; and C is a scaling factor.

To convey the effects of jamming stimuli, we used rectangular pulse trains (figure 1b). The IPIs and SPLs of the imposed jamming stimuli were then characterized by deriving the IPIs and amplitudes of those rectangular pulses. Each rectangular pulse lasted 5 ms. We adjusted the scaling factor for low-TF and high-TF stimuli depending on the behavioural experiments' results obtained here; these *C*-value factors were used to model the frequency-shifting behaviour in group flight. Random fluctuations in the TF were added to the TF of each bat. We applied the Runge–Kutta fourth-order method to solve the above equation, using a 1 ms step. For each stimulus condition, we ran the simulation 20 times.

### (h) Modelling the frequency-shifting behaviour in group flight

We generated flight trajectories of bats in each group for 15 s in a confined 2D space to simulate our behavioural experiments in the experimental chamber (supplementary material, methods).

When bats were affected by each other (i.e. $E_{i}(t)=\;0$ in the equations below) from 5 to 10 s of the 15 s flight period, the flight was designated a group flight. Accordingly, the periods before and after a group flight, when they did not affect each other, were defined here as single flights 1 and 2, respectively. The baseline frequency for each bat in a group was sampled from a normal distribution with a mean of 48 kHz and a standard deviation of 0.4 kHz determined from a previous study [10]. The IPIs of each bat were sampled from a gamma distribution having a mean of 70 ms and one standard deviation of 18.7 ms (supplementary material, figure S4a), which corresponded to the reported values during single flight (63 and 82 ms, respectively) in the previous studies [10,28].

The modelled bats were assumed to emit pulses in their flight direction and at a constant SPL. The SPLs received by a bat from other bats during the group flight were calculated by considering attenuation due to the spatial relationships between individuals. The spreading loss and atmospheric attenuation [40] were calculated based on the distances between the bats (supplementary material, figure S4b,c). We used 50 kHz for calculations of all frequency-dependent attenuations and directionalities, since no marked differences were observed in the directionality of five different frequencies ranging from 48 to 52 kHz (supplementary material, figure S5).

We then built a model for frequency-shifting behaviour in the group flight of bats, using these equations:

$$\begin{array}{c} \tau \;\frac{dF_{i}(t)}{dt}=F_{i0}\;-F_{i}(t)+E_{i}(t)+N(0,\sigma^{2})\\ E_{i}(t)=\underset{j\neq i}{\sum }CP_{j}(t)A_{ij}\\ A_{ij}(t)=\{\begin{array}{clcl} & 1+FD_{ij}/3,\; & \; & -3<FD_{ij}\leq 0\\ & 1-\frac{FD_{ij}}{3},\; & \; & 0<FD_{ij}<3\\ & 0\; & \; & otherwise, \end{array} \end{array}$$

where τ is the leak time constant (200 ms); $F_{i0}$ is the baseline TF of the *i*-th bat; $F_{i}(t)$ is the TF of the *i*-th bat at time t; $N(0,\sigma^{2})$ denotes the random changes in the TF, by sampling from a normal distribution; $E_{i}(t)$ is a jamming effect from other bats on the *i*-th bat; $P_{j}(t)$ is the normalized power of pulses received by the *i*-th bat from other bats; and C is a scaling factor, which changes depending on whether the difference between own TF is higher or lower than those of others. In the present study, C was set to −4 and 20 when a bat TF was lower or higher than that of another bat, respectively. The *C* values were empirically decided from preliminary runs of the simulations to reproduce bi-directional but asymmetrical shifts in TFs observed in the present and previous studies [10,28]. The $A_{ij}(t)$ term is the factor that determines how the *i*-th bat is affected by the *j*-th bat, and $FD_{ij}$ is the frequency difference between *i*-th and *j*-th bats (supplementary material, figure S4d). We performed 100 simulations, using a time step of 1 ms, in MATLAB 2021b software. We reported 95% confidence intervals (95% CIs) for the differences in mean TF as well as means ± s.d.

### (i) Estimating the effect of jamming avoidance responses on echolocation in a group

For the simulations of group flight, we estimated possible numbers of misdetections sensed by each animal. Based on the results of each simulation, we generated FM signals mimicking bat pulses (figure 5a–d) based on a formula that captures an overall spectro-temporal shape of echolocation pulses of *M. fuliginosus* [22,23]. The bandwidth and the duration of these signals were fixed at 40 kHz and 3 ms, respectively, and their amplitudes were normalized. Therefore, the only difference among signals was their TF. We then calculated cross-correlation functions for a pulse of each bat *vis-à-vis* the received pulses from other co-flying bats. Signals from other bats were detected when the peaks of the cross-correlation function corresponding to the other bats' sound exceeded half the maximum value (0.5). We then counted the number of detected pulses from others per emission event. Finally, we considered the single flights in the simulation as group flights without JARs, and conversely the group flights as group flights with JARs. The threshold of 0.5 for sound detection was chosen based on previous findings that range discrimination performance in echolocating bats matches well with the half-width of the envelope of the autocorrelation function of emitted pulses [62]. We also confirmed that the qualitative trend remained unchanged across a range of threshold values (0.3–0.9).

## Supplementary Material



## Data Availability

The data and analysis scripts supporting the findings of this study are available from Figshare [63]. Supplementary material is available online [64].
