# Supplementary material for: Self-organized spectral adjustment in echolocation sounds within group-flying bats
Source: Proc Biol Sci. 2026 Oct 14;293(2080):20260921. doi: 10.1098/rspb.2026.0921 (PMC13678626; doi:10.1098/rspb.2026.0921)
Supplement: Supplementary file 1 [file rspb.2026.0921.zip › rspb.2026.0921/rspb20260921_si_001.docx]

**Supplementary material**

**Paper title:** Self-organized spectral adjustment in echolocation sounds within group flying bats

**Author information:**

Kazuma Hase^*^, Tasuku Miyazaki, Seiya Oka, Noriyoshi Senoo, Hiraku Nishimori, Masashi Shiraishi, Ken Yoda, Kohta I Kobayasi, and Shizuko Hiryu^*^

* To whom correspondence should be addressed. Email: [hasek@med.u-toyama.ac.jp](mailto:hasek@med.u-toyama.ac.jp) (K.H.) or [shiryu@mail.doshisha.ac.jp](mailto:shiryu@mail.doshisha.ac.jp) (S.H.)

**Journal:** Proceedings of the Royal Society B

**Article DOI:** https://doi.org/10.1098/rspb.2026.0921

**Fig. S1. Changes in pulse duration of flying bats in the presence of jamming stimuli**. Changes in duration of emitted pulses by bats in response to low TF-jamming stimuli with different IPIs (a, b), high TF-jamming stimuli with different IPIs (c, d), and low TF-jamming stimuli with different SPLs (e, f). Dashed lines and solid lines indicate the individual mean duration and the time course of changes in the population mean of duration, respectively. Colours correspond to different stimulus parameters in a, c, e. Plots and lines show mean changes in duration of each individual in b, d, f.

**Fig. S2. Changes in pulse initial frequency of flying bats in the presence of jamming stimuli**. Changes in the initial frequency (IF) of emitted pulses by bats in response to low TF-jamming stimuli with different IPIs (a, b), high TF-jamming stimuli with different IPIs (c, d), and low TF-jamming stimuli with different SPLs (e, f). Dashed lines and solid lines indicate individual mean duration and the time course of changes in the population mean of IF, respectively. Colours correspond different stimulus parameters in a, c, e. Plots and lines show mean changes in the IF of each individual in b, d, f.

**Fig. S3. Summary of TF shifts in response to different jamming stimuli.** (Top) Schematic diagrams of low-TF stimuli with different IPIs (left) and high-TF stimuli with different IPIs (middle), and low-TF stimuli with different SPLs (right). (Middle) A matrix represents the amount of TF shifts of each individuals in each stimulus condition. Asterisk shows statistical significance (Student’s t test with Bonferroni correction, *P* < 0.05. The new significance alpha level after Bonferroni correction was 0.0014). (Bottom) Example time course of TF change in response to each condition.

**Fig. S4. IPIs’ generation, the directional and distance-dependent attenuation, and function in the model simulation.** (a) The gamma distribution to generate IPIs for the simulations. (b) The directionality of pulses in the simulation. For comparison, we used the directionality of 50 kHz for all the emissions. (c) The effect of atmospheric attenuation in the simulation. Again, for comparison, we set the attenuation to 50 kHz. (d) The shape of the function $A$, which determines how a bat is affected by other bats depending upon frequency difference $FD$.

 **Fig. S5. Directionality of sounds at different frequencies.** Directionality was calculated for frequencies from 48 to 52 kHz, shown in different colours. The red dotted line indicates −6 dB.

**Fig. S6. Examples of the effect of JAR on the cross-correlation function.** (a–h) Example spectrograms (top) and cross-correlation functions (bottom) of a bat’s pulse vis-à-vis other bats’ pulses received by the bat in a group size of 4 (a), 8 (c), 16 (e), and 32 (g) bats without JAR (left column) and with JAR (right column). The dashed line in the cross-correlation function represents the threshold for detecting pulses. When pulses from other bats exceed that threshold, the pulses are considered as misdetected (indicated by asterisks). The number of pulses crossing the threshold is smaller in the results with JAR than without JAR employed by bats.
