# Supplementary material for: Self-organized spectral adjustment in echolocation sounds within group-flying bats
Source: Proc Biol Sci. 2026 Oct 14;293(2080):20260921. doi: 10.1098/rspb.2026.0921 (PMC13678626; doi:10.1098/rspb.2026.0921)
Supplement: Supplementary file 1 [file rspb.2026.0921.zip › rspb.2026.0921/rspb20260921_si_002.docx]

**Supplementary methods**

**Paper title:** Self-organized spectral adjustment in echolocation sounds within group flying bats

**Author information:**

Kazuma Hase^*^, Tasuku Miyazaki, Seiya Oka, Noriyoshi Senoo, Hiraku Nishimori, Masashi Shiraishi, Ken Yoda, Kohta I Kobayasi, and Shizuko Hiryu^*^

* To whom correspondence should be addressed. Email: [hasek@med.u-toyama.ac.jp](mailto:hasek@med.u-toyama.ac.jp) (K.H.) or [shiryu@mail.doshisha.ac.jp](mailto:shiryu@mail.doshisha.ac.jp) (S.H.)

**Journal:** Proceedings of the Royal Society B

**Article DOI:** <https://doi.org/10.1098/rspb.2026.0921>

## **Methods**

## *Modelling the frequency-shifting behaviour in group flight*

## Because flight of *M. fuliginosus* in the experimental chamber change dynamically in the horizontal plane but not as dramatically as in the vertical plane, present simulations focused on flight trajectories in the two-dimensional (2D) horizontal plane. To reproduce circular flight paths of *M. fuliginosus* we typically observed in the laboratory flight room [64], we set up a bounded 2D space in which an impulsive force from the boundary kept the modelled bats within the simulated flight area. We generated flight trajectories of bats in each group for 15 sec in a the 2D space, by using the following equations to keep the bats in a confined space to simulate our behavioural experiments in the experimental chamber:

##

$$\frac{d\boldsymbol{x}_{i}(t)}{dt}=\boldsymbol{v}_{i}\left( t \right)$$

##

$$m_{i}\frac{d\boldsymbol{v}_{i}(t)}{dt}=\left( \alpha-\left| \boldsymbol{v}_{i}\left( t \right) \right| \right)\boldsymbol{v}_{i}\left( t \right)+\sum_{j\neq i} \boldsymbol{F}_{ij}(t)+\boldsymbol{F}_{wall}(t)+\boldsymbol{R}_{i}(t)$$

## where$\boldsymbol{x}_{i}$ is the two-dimensional position of each *i*-th bat; $\boldsymbol{v}_{i}$ is the two-dimensional velocity of each *i*-th bat, $m_{i}$ is a mass of each *i*-th bat, and $\alpha$ is self-propulsion. The term $\boldsymbol{F}_{ij}(t)$ denotes the repulsive forces acting on each bat from other individuals in the group, $\boldsymbol{F}_{wall}(t)$ is the repulsive force from the nearest wall, and $\boldsymbol{R}_{i}(t)$ is a random factor.

64.	Hase K, Sugihara S, Oka S, Hiryu S. 2021 Absence of Jamming Avoidance and Flight Path Similarity in Paired Bent-Winged Bats, *Miniopterus Fuliginosus*. *Journal of Robotics and Mechatronics* **33**, 564–571. (doi:10.20965/jrm.2021.p0564)
